# Segmental dataset and whole body expression data do not support the hypothesis that non-random movement is an intrinsic property of Drosophila retrogenes

**DOI:** 10.1186/1471-2148-12-169

**Published:** 2012-09-05

**Authors:** Maria D Vibranovski, Yong E Zhang, Claus Kemkemer, Nicholas W VanKuren, Hedibert F Lopes, Timothy L Karr, Manyuan Long

**Affiliations:** 1 Department of Ecology and Evolution, University of Chicago, Chicago, IL 60637, USA; 2 Key Laboratory of the Zoological Systematics and Evolution, Institute of Zoology, Chinese Academy of Sciences, Beichen West Road, Chaoyang District, Beijing, 100101, People’s Republic of China; 3 Committee on Genetics, Genomics, and Systems Biology, University of Chicago, Chicago, IL, 60637, USA; 4 University of Chicago Booth School of Business, Chicago, IL, 60637, USA; 5 Center for Evolutionary Medicine and Center for Infectious Diseases and Vaccinology, The Biodesign Institute, Arizona State University, Tempe, AZ, 85287, USA; 6 Current address: Departamento de Genética e Biologia Evolutiva, Instituto de Biociências Universidade de São Paulo, São Paulo, SP 05508-090, Brazil

## Abstract

**Background:**

Several studies in *Drosophila* have shown excessive movement of retrogenes from the X chromosome to autosomes, and that these genes are frequently expressed in the testis. This phenomenon has led to several hypotheses invoking natural selection as the process driving male-biased genes to the autosomes. Metta and Schlötterer (BMC Evol Biol 2010, 10:114) analyzed a set of retrogenes where the parental gene has been subsequently lost. They assumed that this class of retrogenes replaced the ancestral functions of the parental gene, and reported that these retrogenes, although mostly originating from movement out of the X chromosome, showed female-biased or unbiased expression. These observations led the authors to suggest that selective forces (such as meiotic sex chromosome inactivation and sexual antagonism) were not responsible for the observed pattern of retrogene movement out of the X chromosome.

**Results:**

We reanalyzed the dataset published by Metta and Schlötterer and found several issues that led us to a different conclusion. In particular, Metta and Schlötterer used a dataset combined with expression data in which significant sex-biased expression is not detectable. First, the authors used a segmental dataset where the genes selected for analysis were less testis-biased in expression than those that were excluded from the study. Second, sex-biased expression was defined by comparing male and female whole-body data and not the expression of these genes in gonadal tissues. This approach significantly reduces the probability of detecting sex-biased expressed genes, which explains why the vast majority of the genes analyzed (parental and retrogenes) were equally expressed in both males and females. Third, the female-biased expression observed by Metta and Schlötterer is mostly found for parental genes located on the X chromosome, which is known to be enriched with genes with female-biased expression. Fourth, using additional gonad expression data, we found that autosomal genes analyzed by Metta and Schlötterer are less up regulated in ovaries and have higher chance to be expressed in meiotic cells of spermatogenesis when compared to X-linked genes.

**Conclusions:**

The criteria used to select retrogenes and the sex-biased expression data based on whole adult flies generated a segmental dataset of female-biased and unbiased expressed genes that was unable to detect the higher propensity of autosomal retrogenes to be expressed in males. Thus, there is no support for the authors’ view that the movement of new retrogenes, which originated from X-linked parental genes, was not driven by selection. Therefore, selection-based genetic models remain the most parsimonious explanations for the observed chromosomal distribution of retrogenes.

## Background

In *Drosophila*, there is an excess of retrogenes moving from the X chromosome to autosomal regions [1]. Interestingly, those retrogenes are frequently expressed in testis [1]. Both observations have been reported several times in *Drosophila melanogaste*r [1-3], as well as in other species of mammals [4] and mosquitoes [5,6]. In addition, a comparative study between the genomes of twelve *Drosophila* species revealed excessive movement out of the X chromosome for both retrogenes and DNA-based duplications in the *Drosophila* genus [7,8]. Further, older genes that originated before the split of the *Drosophila* and *Sophophora* subgenera and for which expression is greater in males than females, are under-represented on the X chromosome [9-12]. The gene movement off the X chromosome likely contributed, along with other mechanisms, to the paucity of X-linked male-biased genes found in *Drosophila*[11].

Several hypotheses have been proposed to explain the excessive movement of genes out of the X chromosome and the paucity of male-biased X-linked genes [1,13-19]. These hypotheses include (i) meiotic sex chromosome inactivation (MSCI), (ii) dosage compensation, (iii) meiotic drive, and (iv) sexual antagonism, and they all assume that natural selection has favoured accumulation of male-biased genes on the autosomes [1,13-19]. Two of those hypotheses, MSCI and dosage compensation, have been tested and shown to play a role in the genomic relocation of retrogenes expressed in testis [15,16,20]. MSCI is predicated on the hypothesis that retrogenes located on autosomes continue functioning during male meiosis whereas otherwise they would be subjected to inactivation [1,17,20]. Indeed, in meiosis where MSCI occurs, autosomal retrogenes have higher expression than their parental X-linked genes, presumably to compensate for their inactivation [20]. In *Drosophila*, the dosage compensation hypothesis also predicts a decrease in the number of male-biased genes in the X chromosome relative to autosomes [15,16]. Up-regulation in males is less effective for X-linked genes since they are already hypertranscribed during dosage compensation [15,16]. Consistent with this hypothesis, autosomal retrogenes are often derived from X-linked parental genes that reside close to components of the dosage compensation machinery [16].

The recent study by Metta and Schlötterer [21] proposed a new interpretation which negated the need for selection-based hypotheses to understand the out-of-the X movement pattern of *Drosophila* retrogenes. To test the general role of natural selection, Metta and Schlötterer [21] identified retrogenes for which the parental gene has been lost or degenerated. In other words, the parental genes and retrogenes are never found in the same species. This innovative approach differed from previous studies that analyzed both parental and retrogene copies of the same species [1-3]. A key argument used for their analysis was that the remaining retrogenes assumed and maintained parental ancestral function(s) [21]. This unique set of parental genes and retrogenes (Table 1) displayed no differences in their patterns of DNA sequence evolution nor in sex-biased expression. However, these retrogenes still showed excessive movement out of the X chromosome suggesting no selection for these genes based on differential gene expression in males. Moreover, the genes studied by Metta and Schlötterer [21] displayed female-biased or unbiased (non-sex-biased) expression profiles. Therefore, the authors suggest that such gene movement in *Drosophila* is not related to male-biased expression and therefore is a general non-adaptive property of retrotransposition [21]. 

**Table 1:** **Reproduced from Table 2 in [**[21]

	**Dsim**	**Dyak**	**Dana**	**Dpse**	**Dvir**	**Dmoj**
CG1164	−0.396*	−0.214	−0.366	−0.369	**−0.431**	**−0.449**
CG11790	0.652	−0.198	−0.139	−1.116*	**−0.209**	−0.822*
CG12375	**0.062**	**−0.065**	**−0.248**	−0.486	−0.181	0.077
CG1354	−0.471*	−0.597*	−0.358*	−0.629*	−0.998*	−0.636*
CG14286	**0.378**	**−0.505**	-	0.076	−0.457	−0.961*
CG14618	−0.184	−0.112	−0.064	0.000	**−0.149**	−0.431*
CG14779	−0.036	−0.176	**0.116**	−0.244	−0.151	−0.915
CG1639	−0.052	−0.323	0.019	**−0.402**	0.358*	−0.177
CG16771	**0.414**	**0.163**	**−0.043**	-	−0.082	0.037
CG2059	0.251	0.130	**0.039**	0.181	0.154	0.137
CG2227	−0.151*	**0.128**	**−0.130**	**−0.085**	−0.212	-
CG32441	0.713	0.432	0.002	−0.368*	0.587*	**−0.082**
CG33250	−0.068	−0.321	**−0.071**	−0.286	-	-
CG4918	−0.807*	−0.772*	−1.911*	−2.491*	−1.284*	-
CG5029	0.937*	0.349*	−0.550*	−0.361*	**0.140**	0.615*
CG6284	**−0.216**	**−0.223**	**0.040**	-	−0.214	−0.622*
CG8239	-	−0.298	-	-	**0.044**	**−0.323**
CG8939	−0.512*	−0.449*	**−0.398**	−1.489*	−0.401	−0.271
CG9126	-	−0.003	**−0.017**	-	-	-
CG9172	**0.112**	0.331*	−0.842*	−0.610*	−0.164	0.245
CG9742	−0.577	−0.727*	−0.356	−0.618*	−0.258	−0.915*

Sex-biased gene expression of the genes based on microarray analysis in different *Drosophila* species [9].

Dsim: *D. simulans*; Dyak: *D. yakuba*; Dana: *D. ananassae*; Dpse: *D. pseudoobscura*; Dvir: *D. virilis*; Dmoj: *D. mojavensis*. The expression values are the log_2_ ratios of male vs. female intensities. Negative values indicate that the expression is biased towards females while positive values indicate the opposite. * indicates a significant sex bias in gene expression. Retroposed copies are indicated by bold font.

We revisited the analyses and sex-biased expression data presented by Metta and Schlötterer [21] and found several issues with the retrogene dataset and expression data used that tended to render their arguments arguable. First, we found that the set of retrogenes was a segmental dataset in which the majority of genes with male-biased expression were excluded. Second, we observed that the general unbiased expression they claimed to exist was actually a consequence of the use of expression data from whole animals. Sex-biased gene expression (particularly male-biased expression) is poorly revealed when RNA is obtained from whole-body samples in comparison to dissected tissues (gonads) [6,7,22]. Third, we found that most of the observed female-biased expression is derived from X-linked parental genes. The dataset provided by Metta and Schlötterer [21] shows an excess of X→A movement and therefore contains a significant number of parental genes that are located on the X chromosome, which is known to be enriched with female-biased genes. Fourth, we analyzed additional gonad expression data that support the evidence that autosomal genes show higher male-related expression than X-linked genes. In the following four sections, we report our analyses of Metta and Schlötterer’s [21] data that led to conclusions different from their previous ones.

## Results

### The segmental dataset underestimated male-biased expression

We analyzed the dataset of positionally relocated genes for 12 *Drosophila* species [23], used by Metta and Schlötterer [21]. Bhutkar *et al.*[23] identified 46 cases of inter-chromosomal retrotransposition for which the parental copy had degenerated or had been lost (Methods). Metta and Schlötterer [21] further filtered the dataset by several criteria such as high coverage between orthologous sequence alignments and intron absence to control the data quality (filtered out 26 cases) [21]. Therefore, for those remaining 20 cases together with a previously identified retrogene (*RplP2*), (herein named the segmental dataset), each of the 12 *Drosophila* species has only one orthologous gene that corresponds either to the parental gene or the retrogene. In Metta and Schlötterer’s study [21], *D. melanogaster* expression was retrieved from FlyAtlas [24] (which is based on comparison of gonad expression).

**Figure 1:**
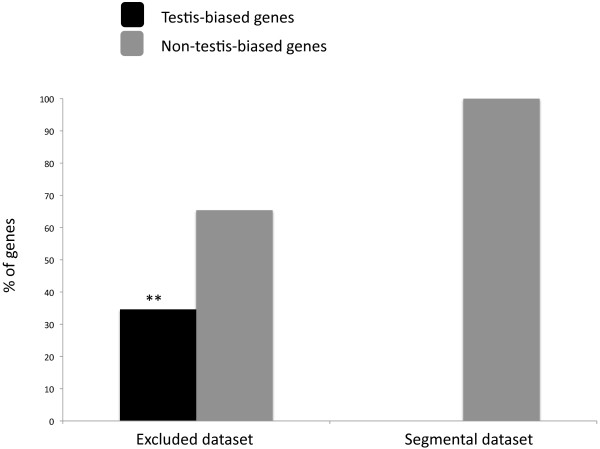
**Percentage of *Drosophila melanogaster *testis-biased and non-testis-biased expressed genes in two different gene expression datasets.** Testis-biased expression profiles for *D. melanogaster* genes were obtained from Metta and Schlötterer [21]. Segmental dataset corresponds to the 21 movement cases selected by Metta and Schlötterer [21] from the original 46 cases in [23]. Excluded dataset corresponds to the remaining 26 cases. The number of testis-biased genes is significantly higher in the excluded dataset (**Fisher exact test, *p* = 0.0025), which implies that the filter used by Metta, and Schlötterer [21] disproportionally selected less testis-biased genes in the segmental dataset.

Metta and Schlötterer [21] found that none of the 21 cases of inter-chromosomal retroposition showed testis-biased expression in *D. melanogaster*. However, the pattern of testis-biased expression changes significantly between the segmental dataset (21 cases) and the initial dataset of 46 retrogenes from Bhutkar *et al.*[23]. Nine out of the 26 remaining cases (herein called the excluded dataset) show testis-biased expression in *D. melanogaster*[21], which is significantly different from the expression patterns found in their segmental dataset (Figure 1, Fisher Exact Test; *P* = 0.0025, Additional file 1). 

Nonetheless, Metta and Schlötterer [21] were aware that the testis expression data limited the analysis to *D. melanogaster* genes (no gonad expression data was available/used for other species). For the cases of retroposition where the parental gene had been lost, the copy present in *D. melanogaster* either corresponded to the parental gene or the retrogene, depending on which species or branch the duplication occurred. Using the segmental dataset and the expression criteria in [25], Metta and Schlötterer [21] found that only one out of five retrogenes located on an autosome is expressed (at very low levels) in the testis, which supported their argument for general female-biased or unbiased expression of retrogenes. However, this result was not consistent in Flyatlas [24] in which three of the five retrogenes (CG14286, CG12375, CG4918) are expressed in testis. Moreover, in the excluded dataset, the only case of an autosomal retrogene (CG10934) in *D. melanogaster* with parental X-linked gene is indeed testis-biased expressed [21].

The difference in sex-biased expression between the excluded and segmental datasets could have compromised their final conclusions [21], as one should expect that data subsets would not show drastic differences in expression patterns. One possibility is that the conservative sequence similarity used to construct the segmental dataset biased their acquisition of male-biased expressed genes since in *Drosophila* this class of genes is known to be more divergent than female-biased or unbiased expressed genes [26,27].

However, the conservation of sequence similarity was not the only threshold used to remove genes from the segmental dataset [21]. Other criteria, such as absence of introns, were also implemented [21]. Therefore, it is possible that the segmental dataset represents an even more confident set of relocated retrogenes. Therefore, we conducted a full analysis on the excluded dataset (26 cases, see Additional file 2). We found no evidence to exclude the following cases: CG32119, CG14077, CG7557, CG8928, CG4904, CG14026 and CG12010. Note that three of those genes are male-biased expressed. Thus, those highly confident relocated genes contained in the excluded dataset still show a significantly higher frequency of male-biased genes than the segmental dataset (3 out of 7 vs. 0 out of 21 or 43% vs. 0%, Fisher Exact Test, p=0.0107). Nonetheless, we focused our further analyses only in the segmental dataset used by Metta and Schlötterer’s [21]. In the following three sections, we present several points that led us to continue to have a different conclusion.

### Whole-body gene expression comparison between males and females underestimated male-biased expression

**Figure 2:**
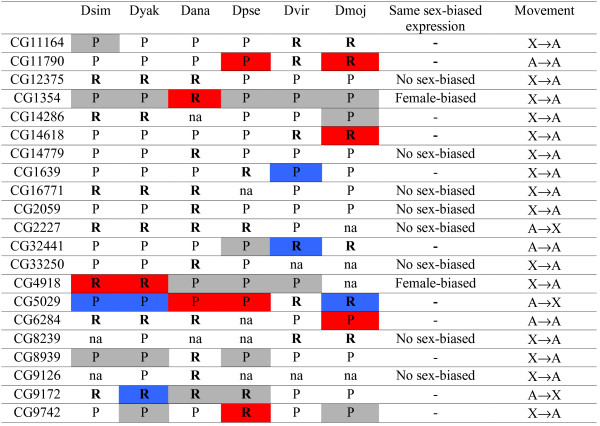
**Significant sex-biased gene expression.** Adapted from Table 2 in [21]. Dsim: *D. simulans*; Dyak: *D. yakuba*; Dana: *D. ananassae*; Dpse: *D. pseudoobscura*; Dvir: *D. virilis*; Dmoj: *D. mojavensis*. Significant female- and male-biased expression is represented in red and blue, respectively. Female-biased expressed genes located on the X chromosome are shown in grey boxes. Retrogenes and parental genes are shown in “R” and “P”, respectively. Same sex-biased expression can be divided in: no sex-biased expression and female-biased expression for all orthologs analyzed. “-” corresponds to cases where orthologos do not show the same sex-biased expression. “na” refers to no expression data available.

\In order to test for functional equivalence among duplicate copies, Metta and Schlötterer [21] compared the sex-biased gene expression between retrogenes and parental copies. They used the available gene expression data from whole body of males and females in *D. simulans*, *D. yakuba*, *D. ananassae*, *D. pseudoobscura*, *D. virilis* and *D. mojavensis*[26] to classify those genes into different categories in terms of sex-biased expression. They found that retrogenes and parental genes usually show similar expression. Indeed, almost 50% (10/21) of cases have the same sex-biased expression across all species (see Table 1 reproduced from Table 2 in [21]). However, our re-analysis of the data (Figure 2, Additional file 1) revealed that approximately 80% of those cases (8 out of 10) with same sex-biased expression show no significant evidence for male- or female-biased expression. Note that we used the same source to obtain information regarding male- or female- biased expression [26] (see methods). All of them are equally expressed among males and females (unbiased expression or “No sex-biased” in Figure 2). Note that our re-analysis has shown that one additional case of relocation (CG2227, Additional file 1) has unbiased expression in *D. simulans*[21,26] .

The sex-biased expression data used by Metta and Schlötterer [21] came from a previously published article that compared whole body expression of males and females [11,26], whereas previous analyses of gene movement with male expression in *Drosophila* utilized expression data from testes and ovaries [1-3]. It was reported that the number of genes with sex-biased expression is drastically reduced in the whole body expression data of *D. melanogaster*[9]. We also have previously observed that analysis of gene duplicates using whole body expression data only recovered 30% of the male-biased gene expression in *D. melanogaster* gonads [7]. This low coverage of male-biased genes in the whole body data was also observed in *Anopheles gambiae*[6,22]. In this case an even smaller proportion of male-biased genes is observed when compared to the proportion of female-biased genes: only 7% of testis-biased expression is recovered using male whole-body RNA. In contrast, 50% of ovary-biased expression is recovered when using whole-body of females [22]. Moreover, the number of female-biased genes can also be underestimated using whole-body RNA. Since those genes are widely expressed [24], the introduction of somatic tissues in the RNA pool may distort the relative excess found in the ovary. Therefore, the use of whole-body RNA underestimates in general detection of sex-biased genes found by gonadal tissue comparisons.

Metta and Schlötterer [21] also claimed that 60% of genes that have heterogeneous sex-biased expression, *i.e.* cases in which orthologs of the same gene in different species have different sex-biased expression. Moreover, they found that sex-biased expression among species show no particular pattern associated with retrogenes or parental copies (Table 1). However, this result is not unexpected as only 11 out of the 41 retrogenes (27%) displayed sex-biased expression for all species/gene combinations (Figure 2). We therefore reason that any conclusions regarding the relationship between sex-biased expression and chromosomal locations of retrogenes without parental genes must await additional studies using comparisons between gonads in males and females (see “Additional gonad expression data supports selection hypothesis for movement out of the X chromosome” below).

### Female-biased expression is associated with X-linkage of parental genes

Metta and Schlötterer [21] claimed that genes in their dataset show a high frequency of female-biased expression in contrast to the male-biased expression usually found for retrogene moving out of the X chromosome. They interpreted this lack of association and the apparent non-random gene traffic off the X to reflect a non-adaptive process. However, we found that this level of female-biased expression (29/116 species/gene combinations, Tables 1 and Figure 2) is a consequence of large number of X-linked parental genes present in the dataset and therefore is not unexpected even under selection-driven models. In other words, there is an excess of the X-linked gene movement to the autosomes in their dataset. If all orthologs to the 21 retrogenes across the twelve *Drosophila* species are analyzed, it is clear that there will be an enrichment of X-linked parental genes when analyzing the total expression profile (80% vs. 20%, n = 119; Fisher’s Exact test, p < 0.0001). As the X chromosome in *Drosophila* is enriched with female-biased genes [9], it is reasonable to expect a high frequency of this class of gene.

Indeed, we found that most of those female-biased genes are parental genes located in the X chromosome where 18 (grey boxes, Figure 2) out of the 27 female-biased genes are located on the X chromosome and only two are retrogenes (Figure 2). Note that two of 29 genes previously found to be female-biased expressed are actually unbiased expressed between males and females (see notes in Additional file 1). In other words, a high frequency of female-biased genes as 60% (16/27) are X-linked parental genes and the X chromosome is known to be enriched with parental genes and female-biased expressed genes [1,9]. This association can be clearly seen as an enrichment of X-linked female-biased genes for parental copies but not for retrogenes (Table 2, Fisher Exact test, p = 0.0061). Removal of female-biased X-linked genes from Table 1 (grey boxes, Figure 2), results in a noticeable decrease in sex-biased expression, particularly for retrogenes: 3 male-biased and 6 female-biased expressed genes. Therefore, the large number of female-biased genes associated with X-linkage of parental genes is expected from various forms of sexual antagonism [13,14,28] models and consistent with the known deficit of male-biased genes on the X chromosome and enrichment of female-biased genes [9,12]. In other words, their finding of excess of female-biased genes is actually in agreement with proposed selection-based hypotheses connected to sex-biased expression [9-14]. 

**Table 2:** Chromosomal distribution of female-biased genes

	**Parental Genes**	**Retrogenes**
X	16 (84%)	2 (25%)
A	3 (16%)	6 (75%)

Percentages are given inside the parentheses.

X: X chromosome; A: Autosomes.

Fisher Exact test, *p* = 0.0061.

### Additional gonad expression data supports selection hypothesis for movement out of the X chromosome

We searched for additional gonad expression data for the specific group of retrogenes and their parental counterparts analyzed by Metta and Schlötterer [21]. If the selection is driving the retrogene movement out of the X chromosome, we should be able to detect lower expression in ovaries and higher expression in testis for those genes located in the autosomes in comparison to X-linked genes. However, if the movement out of the X chromosome is an intrinsic property of the retrogenes, no differences of sex-related expression should be expected.

Although such assessment is not trivial given the small sample size (entire dataset = 47; segmental dataset = 21 [21]), we were able to find significant differences in at least two independent analyses. First, using FlyAtlas [24] expression data for the segmental dataset of *D. melanogaster* (n = 21), we found that parental genes are more up regulated in ovary than retrogenes (Table 3 and Additional file 1, 93% vs. 43%; Fisher exact test, *p* = 0.0251). This pattern is not a result of the great number of X-linked genes found in the group of parental genes as none of the X-linked retrogenes is up regulated in the ovary (Table 3). This is in contrast to the expression profile of X-linked parental genes, which are all up regulated in the female organ (Fisher exact test, *p* = 0.001). 

**Table 3:** **Distribution of genes up regulated in ovary (FlyAtlas**[24]**)**

	**Parental genes**		**Retrogenes**	
	**X**	**A**	**X**	**A**
Up	11 (79%)	2 (14%)	0 (0%)	3 (43%)
None/Down	0 (0%)	1 (7%)	2 (28.5%)	2 (28.5%)

Percentages are given inside the parentheses.

X: X chromosome; A: Autosomes.

Second, using two different spermatogenic expression profiles [20,29], we found that *D. melanogaster* autosomal genes described by Metta and Schlötterer [21] (entire dataset, n = 47) were more likely expressed in meiosis than in mitosis. Additional file 3: Figure S1 plots the correlation between two available expression profiles in *D. melanogaster* spermatogenesis [20,29]. One of the profiles corresponds to the expression fold difference found between *bag-of-marbles* (*bam*) mutant and wild type testes [29]. The *bam* mutation prevents the entry into meiosis stage and results in the accumulation of pre-meiotic cells [30]. The other profile corresponds to the expression fold difference found between mitotic and meiotic cells dissected from wild-type testes [20]. Both expression profiles are significantly correlated and therefore should reproduce the expression differences between the first two phases of spermatogenesis (*r*^2^ = 0.41, *p* = 2.3e-06). In the latter profile [20], the X-linked genes analyzed in Metta and Schlötterer’s [21] sample show a higher mitotic/meiotic expression when compared to genes located in the autosomes (*t*-test = 2.03, *p* = 0.048). This result suggests that autosomal genes are more frequently expressed in meiotic cells of the testis.

These independent analyses have shown that autosomal- and X-linked genes analyzed by Metta and Schlötterer [21] are not equally expressed regarding sex-related tissues: the autosomal genes tend to be less ovary-expressed and tend to show more male expression, more specifically the meiotic phases of the testis. This result is therefore in agreement with the hypothesis that selective forces such as MSCI, dosage compensation and sexual antagonism are involved in the retrogene movement out of the X chromosome [1,13-17]. It is important to notice that the selective model does not necessarily require male-biased expression, but higher male expression of autosomal retrogenes than of their X-linked parental counterparts.

## Discussion

Numerous studies have shown increased testis expression of retrogenes that have moved out of the X chromosome in *D. melanogaster*[1-3,7,8]. Those findings are associated with several evolutionary hypotheses in which autosomal male-biased genes have been favoured by natural selection [1,13-19]. However, the recent study of Metta and Schlötterer [21] found no evidence of male-biased expression among retrogenes for which the parental copy has been lost. On the contrary, the genes analyzed have mostly female-biased or unbiased expression [21]. As those genes also show the excessive movement out of the X chromosome, Metta and Schlötterer [21] suggested that such a trend is an intrinsic property of retrogenes in *Drosophila* and not part of an adaptive process.

The segmental dataset used by Metta and Schlötterer [21] did not show the same proportion of testis-biased expressed genes observed in the entire dataset of retrogenes in which the parental gene was subsequently lost [23]. Thus it is clear that the segmental dataset used by Metta and Schlötterer was not representative of the entire dataset of retrogenes for which the parental copy has been lost and the authors therefore took this as evidence against selection-based hypotheses [21].

In addition, statistical analysis of gene movement and sex chromosome evolution can only be performed using tissue-specific expression profiles across species, particularly male gonads [1-3,6,7,9,20]. However, such studies are complicated in cases where the parental copy has degenerated or has been lost. In those instances, movements of parent and retrogenes can only be inferred using genomic comparisons and phylogenetic inference between different *Drosophila* species [7,8,21,23]. Unfortunately, expression data derived from gonad analysis do not yet exist for all genomic sequenced *Drosophila* species (only whole-body expression data has been assembled in [26]).

Although a previous study of whole-body expression analysis successfully detected the non-random chromosomal distribution of sex-biased genes [11], it failed to recover the known extensive male-biased expression obtained using tissue-specific data in *D. melanogaster*[7]. That means whole-body expression analyses lack the statistical power needed to detect the tissue-specific basis of retrogene movement out of the X chromosome [7,8] probably due to the smaller sample size of this dataset in comparison to genome-wide analyses. In a previous study [7], we approached this problem by using a conservative analysis of gene movement in *D. melanogaster* for which gonad expression data are available [7,24]. Although the number of retrogenes was too small to conduct a statistical test, it was possible to show that X-linked parental genes for which the corresponding retrogene had moved to the autosomes were generally under-expressed in testis in agreement with sexual antagonism, MSCI and dosage compensation models [7]. Thus, hypotheses concerning the generality of retrogene movements from the X (with or without parental genes) cannot be tested with existing expression data. We must await the acquisition of appropriate tissue-specific expression data from across the *Drosophila* clade.

However, we were able to show that there is an association of sex-biased expression with movement out of the X chromosome within the group of retrogenes analyzed by Metta and Schlötterer [21]. First, using *D. melanogaster* gonad data from FlyAtlas [24], we found the X-linked parental genes tend to be more up regulated in ovaries than retrogenes located in the autosomes. Second, autosomal genes tend to more expressed in meiotic cells of the testis in comparison to X-linked genes. Those results are in agreement with the hypothesis that autosomal regions provide a favourable environment for male-expression [1,13-19,31].

Nevertheless, it is important to notice that even if the tissue-specific data across the *Drosophila* clade provides evidence for reduced testis-biased expression of retrogenes without parental genes compared to that of retrogenes with parental copies, it will not necessarily rule out MSCI, sexual antagonism, meiotic drive and dosage compensation models [1,13-19]. The current sex-biased expression of retrogenes without parental gene does not necessary reflects expression levels when duplication occurred. In this model of retrotransposition, it is reasonable to assume that before the parental gene is lost, the retrogene would either complement the parental gene’s function, or undergo neo- or sub-functionalization [21]. Only after degeneration of the parental copy could selection favour mutations in the retrogene that gradually restore the parental function [21]. Therefore, for the selection-driven hypothesis, male-biased expression is only expected by the time the inter-chromosome movements have occurred.

In addition, there are several other lines of evidence supporting hypotheses that predict excessive gene movement off the X chromosome is driven by natural selection. First, the excessive gene movement out of the X chromosome is not exclusively found in retrogenes. Genes created by DNA-based mechanisms also show excessive out-of-the-X movement, which suggest that natural selection, rather than mutation processes intrinsic to retrotransposition, played an essential role in distributing male-biased genes [7,8]. Second, chicken and silkworm, which have ZW sex determining systems, also present association between sex-bias gene expression and chromosomal gene movement. In those cases, a symmetrical pattern to the XY sex determining system is observed: genes that move out of the Z chromosome tend to be ovary-biased expressed [32,33]. Therefore the phenomenon is not dependent on mutational processes intrinsic to the testis expression and therefore is more likely to be driven by natural selection. Third, a recent population genomic analysis of the copy number variants of *Drosophila* retrogenes found that there are more fixed than polymorphic retrogenes originating on the X chromosome, which provided direct and strong population genetic evidence for the positive selection hypotheses [34]. Fourth, it worth mentioning that several autosomal retrogenes that moved out of the *Drosophila* X chromosome showing clear testis-specific functions have been indentified and extensively described. Examples of those genes are *Drosophila nuclear transport factor-2-related* (*Dntf-2r), Rcd-1 related* (*Rcd-1r*) and *gasket* (*gskt*), [1,35-37].

## Conclusions

Our re-analysis of Metta and Schlötterer’s [21] data mainly revealed that whole body expression analyses are unable to accurately assess sex-biased expression of retrogenes. A similar issue has been recently resolved in mosquitoes [5,6]. The association between male-biased expression and *Anopheles gambiae* retrogene movement out of the X chromosome has been obscured by whole body data [5,38], but revealed in experiments using dissected testes [6]. The available evidence argues against Metta and Schlötterer’s [21] results and interpretations, and reanalysis of their data suggests that retrogenes with parental copies do not tend to be female-biased or unbiased in their expression. We therefore conclude that the excessive movement out of the X chromosome is not an intrinsic property of the retrogenes in *Drosophila* but instead the result of selective forces acting on males.

In conclusion, we note that the conclusions of Metta and Schlötterer [21] have been cited by others [39,40]. It is the hope that our reanalysis of their work will serve to re-focus and clarify the importance of biological relevance in database construction and analysis of gene traffic in *Drosophila*. This is a crucial element to move forward in understanding the role of selection-driven hypotheses such as MSCI, dosage compensation, meiotic drive and sexual antagonism in sex chromosome evolution [1,13-19].

## Methods

### Retrogene and parental gene identification

We retrieved the 47 genes analyzed by Metta and Schlötterer [21] from their Additional file 5. Those genes correspond to *D. melanogaster* genes involved in inter-chromosomal retrotransposition for which the parental copy had degenerated or had been lost, previously identified in [23]. Following Metta and Schlötterer’s [21] classification, we separated those 47 inter-chromosomal gene movements into two sub-datasets here named by us as the segmental and the excluded datasets. The former contains 21 cases, which Metta and Schlötterer [21] selected by several criteria in order to control the data quality (see details in Additional file 1). The excluded dataset corresponds to the remaining 26 cases. In order to search for orthologs of the segmental dataset genes in other *Drosophila* species, we used the 21 *D. melanogaster* CGs as Flybase queries [41]. Using the result from genome-wide drosophilid orthologs, we searched for GLEANR identifiers through the FlyBase FBgn-GLEANR ID Correspondence Table. GLEANR identifiers are listed in our Additional file 1.

### Gene expression analysis

For the 21 gene movements presented in the segmental dataset, we searched for sex-biased pattern in male vs. female whole body comparisons in six *Drosophila* species [26]. In order to reproduce expression data from Metta and Schlötterer [21] in non-*D. melanogaster* species, we used the GLEANR identifiers to search for male- and female-biased genes identified in Supplemental Tables 5–16 in [26]. Genes that were not presented in those tables were considered as unbiased expressed genes between males and females.

Testis-biased expression profiles for *D. melanogaster* genes were obtained from Metta and Schlötterer [21] analysis marked in red both in Additional file 1 here and in Additional file 5 in [21]. We re-analyzed the presence of expression in testis for all five retroposed copies in the segmental dataset that are located on the autosomes in *D. melanogaster.* Using the Affymetrix present call classification in FlyAtlas (4 out of 4 arrays), we observed that 3 out of the 5 retrogenes are expressed in testis in *D. melanogaster* as opposed to only one described in [21]. *D. melanogaster* up regulation in ovary or testis in comparison to the whole body was also obtained from FlyAtlas [24] and is described in Additional file 1, Additional expression sheet.

Expression data on specific stages of *D. melanogaster* spermatogenesis was obtained from both *bam* mutant whole testes and from mitotic and meiotic phases of wild-type testes [20,29]. Normalized expression data for the 47 *D. melanogaster* genes involved in gene movement were obtained from Tables S1 in [20,29] by crosslinking Oligo identifiers and are described in Additional file 1.

### Statistical Analysis

In-house Perl scripts and unix commands were used to analyze different groups of data. Significances of the differences in 2x2 contingency tables were always assessed with Fisher’s exact tests as implemented in R.

## Authors’ contributions

MDV, TLK, and ML conceived the study. MDV, YEZ and NWV performed the computational experiments. MDV, YEZ, CK and NWV analyzed the data. HFL developed the statistical model. HFL and MDV performed the statistical analyses. MDV, TLK and ML collated, assembled and, with assistance and approval of all authors, wrote the manuscript.

## Supplementary Material

Additional file 1 **List of Retrogenes and their sex-biased information.** Modified from Additional file 5 in Metta and Schlotterer [21]. Sex-biased and spermatogenic expression and movement direction for candidate genes were obtained from [11,20,21,24,26,29]. Click here for file

Additional file 2 Detail analysis on the 26 relocated cases contained in the excluded dataset. Click here for file

Additional file 3 **Figure S1.** Correlation between two expression datasets from *Drosophila* spermatogenesis [1,2]. X-axis represents the fold differences between bam mutant and wild type testis from [1]. Y-axis represents the fold differences between mitotic and meiotic expression of spermatogenesis from [2]. Forty-seven *D. melanogaster* genes analyzed by Metta and Schlötterer [3] were plotted (*r*^2^ = 0.41; *t*-test for regression, *t* = 30.07, *p* = 2.3e-06). The segmental dataset selected by the same group [3] (21 genes) also presents a similar pattern of correlation. X- and Autosomal-linked genes are shown in green and red, respectively. Average fold difference between mitotic and meiotic expression for X-linked genes are higher than for genes located in the autosomes (0.48 vs −0.06; *t*-test = 2.03, *p* =0.048. Click here for file
