# Supplementary material for: Segmental dataset and whole body expression data do not support the hypothesis that non-random movement is an intrinsic property of Drosophila retrogenes
Source: BMC Evol Biol. 2012 Sep 5;12:169. doi: 10.1186/1471-2148-12-169 (PMC3532075; doi:10.1186/1471-2148-12-169)
Supplement: Additional file 1 — List of Retrogenes and their sex-biased information. Modified from Additional file 5 in Metta and Schlotterer [21]. Sex-biased and spermatogenic expression and movement direction for candidate genes were obtained from [11,20,21,24,26,29]. [file 1471-2148-12-169-S1.doc]

**
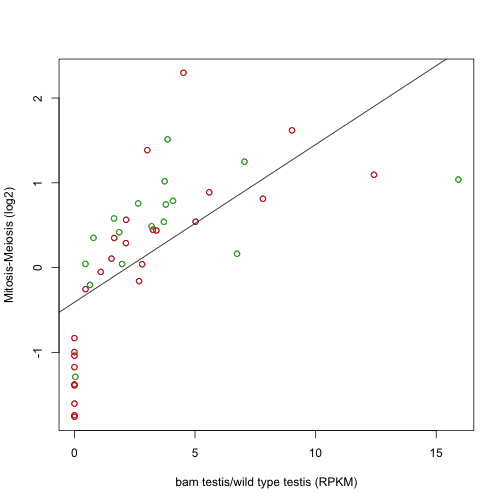
**

**Figure S1.** Correlation between two expression datasets from *Drosophila* spermatogenesis [1,2]. X-axis represents the fold differences between bam mutant and wild type testis from [1]. Y-axis represents the fold differences between mitotic and meiotic expression of spermatogenesis from [2]. Forty-seven *D. melanogaster* genes analyzed by Metta and Schlötterer [3] were plotted (*r*^2^ = 0.41; *t*-test for regression, *t* = 30.07, *p* = 2.3e-06). The segmental dataset selected by the same group [3] (21 genes) also presents a similar pattern of correlation. X- and Autosomal-linked genes are shown in green and red, respectively. Average fold difference between mitotic and meiotic expression for X-linked genes are higher than for genes located in the autosomes (0.48 vs -0.06; *t*-test = 2.03, *p* =0.048).

References

1. Gan Q, Chepelev I, Wei G, Tarayrah L, Cui K, Zhao K, Chen X: **Dynamic regulation of alternative splicing and chromatin structure in Drosophila gonads revealed by RNA-seq.** *Cell Res.* 2010 20(7):763-783.
2. Vibranovski MD, Lopes HF, Karr TL, Long M: **Stage-specific expression profiling of *Drosophila* spermatogenesis suggests that meiotic sex chromosome inactivation drives genomic relocation of testis-expressed genes.** *PLoS Genet* 2009, **5:**e1000731.
3. Metta M, Schlötterer C: **Non-random genomic integration - an intrinsic property of retrogenes in Drosophila?** *BMC Evol Biol* 2010, **10:**114.
