# Supplementary material for: Segmental dataset and whole body expression data do not support the hypothesis that non-random movement is an intrinsic property of Drosophila retrogenes
Source: BMC Evol Biol. 2012 Sep 5;12:169. doi: 10.1186/1471-2148-12-169 (PMC3532075; doi:10.1186/1471-2148-12-169)
Supplement: Additional file 2 — Detail analysis on the 26 relocated cases contained in the excluded dataset. [file 1471-2148-12-169-S2.doc]

We conducted a full analysis on the excluded 26 cases (see details below), we found no evidence to exclude the following cases: CG32119, CG14077, CG7557, CG8928, CG4904, CG14026 and CG12010. It occurs that these cases should be included in the analysis. We see that three of those genes are male-biased. Thus, the excluded dataset has a significantly higher frequency of male-biased genes than the selected 21 cases (43% vs. 0%, Fisher Exact Test, p=0.0107).

Case by case analysis (n=26):

1) CG10934

For Bhutkar et al [1], the candidate orthologous gene found in the outgroups like *D. virilis* has weak similarity with CG10934. For example, between GJ15731 and CG10934, the evalue is approximately 0.1. However, GJ15731 is as long as 962 AAs, while CG10934 is only 194 AAs suggesting these two genes may not be the real orthologs.

2) CG5509

Ensembl tree building shows that CG5509 maps to the candidate ortholog (GK18217) of *D. willistoni*. The blast evalue is 1e-21 with ID of 31% and the length is also similar suggesting they are indeed orthologs. Possible ortholog exists for other outgroups. For example, the region scaffold_12855:5557068-5557529 of *D. virilis* appears to encode a gene, which is similar to CG5509, although Flybase does not annotate it out. From these evidence, this may be a real relocation case, although whether it happened in the DNA or the RNA-level is difficult to infer given both CG5509 and GK18217 are single-exon genes.

3) CG7557

This gene is shared across all 12 species with orthologous genes encoded by *D. pseudoobscura*/*D. persimilis* relocated in this lineage. The candidate-relocated gene encoded by *D. pseudoobscura* (GA27176) is annotated by flybase as single-exon gene. Metta and Schlötterer [2] argued that this gene may be false annotated and it should consist of three exons based on Genewise predication with modified parameters. Flybase annotations showed that 12 out of 13 predication tools inferred one exon in this region including genewise with default parameters, which generated a single continuous high-quality alignment for majority of coding regions of CG7557. As supporting evidence, UCSC genomic alignments showed that *D. pseudoobscura*/*D. persimilis* does not share both GT-AG splicing junctions encoded by *D. melanogaster*. With modified parameters, gene models predicting three exons could be predicted. However, the two introns are not orthologous to those encoded by *D. melanogaster*. In the addition, the flanking regions are poorly aligned.

Thus, GA27176 may be more likely to be single-exon gene rather than a gene with three exons. In other words, there is no strong reason to reject this candidate retroposition event.

4) CG13330

From Flybase's website, indeed this gene together with flanking three genes relocated together suggesting that this is not a real retroposition event.

5) CG13762

This case indeed has some problem. Basically, Bhutkar et al [1] did not find an ortholog in *D. grimshawi*, *D. mojavensis* and *D. virilis*. However, this may be wrong. For example, in *D. grimshawi*, GH14404 appears to be a best-to-best ortholog of CG13762 based on UCSC genomic alignment. GH14404 is in the Muller D element. Thus case is more likely to be a one-exon-to-multiple-exon DNA-level D-A relocation event.

6) CG14077

This case is similar to CG7557. The major-voting prediction is a single-exon gene in the orthologous region of *D. pseudoobscura*. GT-AG splicing junctions are not shared between *D. melanogaster* and D. pseudoobscura. So, there is no strong reason to reject this case. With default parameters, genewise predict a single-continuous alignment, i.e., single-exon gene. With the global alignment option, it predicts one intron. However, the flanking region of this intron does not align very well suggesting this intron may not be that reliable. Moreover, based on the location of this candidate intron, it is not shared by *D. melanogaster*. And all three introns encoded by *D. melanogaster* could not be predicted by genewise even with modified parameters. All together, this case should be viewed as a retroposition.

7) CG31752

This case is ambiguous. This gene has candidate homolog in D. willistoni and *D. virilis* with blastp evalue < 1e-20. However, in *D. pseudoobscura*, the top three candidate hits are all around 1e-10 by tblastn search. It is difficult to infer which one is the real ortholog given such a high divergence.

8) CG31773

Gene prediction tools get in conflict for this locus. Some tools support the existence of intron in *D. pseudoobscura*, while the others do not.

9) CG32119

This case is similar to CG14077 and CG7557. Namely, the major-voting prediction is a single-exon gene in the orthologous region of *D. pseudoobscura*. The GT-AG splicing junction is not shared beween *D. melanogaster* and *D. pseudoobscura*. In addition, Genewise with default parameters generate a single continuous alignment. With the global option, genewise can indeed predict an intron. However, the intron together with the flanking region is poorly aligned compared to the other region suggesting this part may not be truly homologous. Moreover, based on the location of this candidate intron, it is not shared by *D. melanogaster*. The single intron encoded by *D. melanogaster* could not be predicted by genewise even with modified parameters. All together, this case is more likely to be a retroposition.

10) CG16775

It is subject to multiple rounds of duplication in the drosophila subgroup. It is difficult to infer true orthology. So, it is better to be excluded.

11) CG10555

The orthologous locus in *D. simulans* is not fully assembled. Thus, the gene model is not predicted in flybase. However, there is indeed a duplicate in 3R. So, this appears not a relocated gene, but a duplicated gene.

12) CG7313

This case is similar to CG10555 with the real ortholog of CG7313 not assembled in *D. simulans*.

13) CG9504

The candidate ortholog in *D. simulans* (GD22251) is actually a chimeric gene generated by DNA-level relocation of CG9504 together with its neighboring gene CG9503.

14) CG8928

This case is similar to CG32119. Considering the genomic alignment around GT/AG, the gene prediction results and the location of candidate intron in *D. ananassae*, the intron-containing gene model is possibly untrue. So, this case should not be excluded.

15) CG8231

Yes, this is a retroposed duplication.

16) CG11093

The candidate ortholgous locus in *D. ananassae*, GF19850, is encoded by scaffold_13335. Although the local region of GF19850 is difficult to determine its linkage, the majority of scaffold_13335 maps to X of *D. melanogaster*, *i.e.*, Muller element A. However, the introns predicted by genewise with modified parameters are indeed consistent with *D. melanogaster*. So, this relocation may have happened on DNA level.

17) CG3510

This case is similar to CG10555 with the orthologous locus in Muller element C not fully assembled.

18) CG3893

Indeed, in the possibly orthologous region of *D. persimilis*, there is a big sequencing gap.

19) CG3917

Similar to CG8231, the retroposed duplication occurred in *D. grimshawi*.

20) CG4904

The local region of candidate *D. willistoni* ortholog is indeed difficult to infer in the muller arm. However, taking the contig as a whole, for genes with *D. melanogaster* orthologs, most of them do map to 2R of *D. melanogaster*, namely Muller element C.

GT/AG is conserved from *D. melanogaster* to *D. grimshawi* for both introns, but not for *D. willistoni*. And prediction tools support intronless gene models. However, genewise does predict one intron, which concurs with the location of the orthologous intron in *D. melanogaster*, while the other one intron could not be predicted.

Therefore, it seems that relocation does occur in *D. willistoni*. It may be a retroposition of one partially spliced mRNA.

21) CG4908

*D. willistoni* ortholog annotation has been removed in flybase. Also, *D. melanogaster* gene model does have one intron.

22) CG13576

Due to the exitence of nearby sequencing gap, it is difficult to infer whether intron loss occurred.

23) CG14026

GT/AG is conserved from *D. melanogaster* to *D. grimshawi*, but not for *D. virilis* and *D. mojavensis.* Moreover, the prediction tools support intronless gene models. Even with modified genewise parameters, only one intron could be predicted, while the other two could not be predicted. Moreover, the gene size is up to 50 kb in *D. melanogaster* (depending on different transcripts), while the orthologous locus is only like 5kb in *D. virilis* and *D. mojavensis.* Overall, this should be a retroposition or at least a retroposition of partially spliced transcript.

24) CG12010

*D. melanogaster* GT/AG is not shared by drosophila subgroup. The prediction tools support intronless gene models. Moreover, local/global, 2123/623 parameters in genewise, failed to generate a intron-containing gene models in *D. grimshawi* with CG12010-PB as the protein template and GH11210 as the DNA locus.

25) CG4694

Taking *D. grimshawi* as an example, the best hit is actually GH17467, which does have one intron predicted.

26) CG7014

Ensembl tree building information indicates that *D. willistoni* encodes three homologs, which appear to be more similar to rps5a (CG8922) rather than rps5b (CG7014). In other words, true orthology may be difficult to infer for this case.

References

1. Bhutkar A, Russo SM, Smith TF, Gelbart WM: **Genome-scale analysis of positionally relocated genes.** *Genome Res.* 2007, **17:**1880–1887.

2. Metta M, Schlötterer C: **Non-random genomic integration - an intrinsic property of retrogenes in Drosophila?** *BMC Evol Biol* 2010, **10:**114.
